# Pro- and Anti-Tumoral Factors Involved in Total Body Irradiation and Interleukin-2 Conditioning in Adoptive T Cell Therapy of Melanoma-Bearing *Rag1* Knock-Out Mice

**DOI:** 10.3390/cells11233894

**Published:** 2022-12-02

**Authors:** Seon-Hee Kim, Eun Mi Go, Dong Hoon Shin, Beom K. Choi, Chungyong Han

**Affiliations:** 1 Immuno-Oncology Branch, Division of Rare and Refractory Cancer, Research Institute, National Cancer Center, Goyang 10408, Republic of Korea; 2 Department of Biomedical Laboratory Science, Catholic Kwandong University, Gangneung 25601, Republic of Korea; 3 Targeted Therapy Branch, Division of Rare and Refractory Cancer, Research Institute, National Cancer Center, Goyang 10408, Republic of Korea; 4 Department of Cancer Biomedical Science, Graduate School of Cancer Science and Policy, National Cancer Center, Goyang 10408, Republic of Korea; 5 Biomedicine Production Branch, Cancer Immunotherapy Working Group, Research Institute, National Cancer Center, Goyang 10408, Republic of Korea

**Keywords:** adoptive T cell therapy, total body irradiation, interleukin-2, melanoma, *Rag1* knock-out mice, dendritic cell

## Abstract

In adoptive T cell therapy (ACT), the transfer of tumor-specific T cells is paralleled by the conditioning regimen to increase therapeutic efficacy. Pre-conditioning depletes immune-suppressive cells and post-conditioning increases homeostatic signals to improve the persistence of administered T cells. Identifying the favorable immunological factors involved in a conditioning regimen is important to design effective strategies in ACT. Here, by using an ACT model of murine melanoma, we evaluate the effect of the total body irradiation (TBI) and interleukin-2 (IL-2) treatment combination. The use of a *Rag1* knock-out strain, which lacks endogenous T cells, enables the identification of factors in a way that focuses more on transferred T cells. We demonstrate that the TBI/IL-2 combination has no additive effect in ACT, although each conditioning improves the therapeutic outcome. While the combination increases the frequency of transferred T cells in lymphoid and tumor tissues, the activation intensity of the cells is reduced compared to that of the sole TBI treatment. Notably, we show that in the presence of TBI, the IL-2 treatment reduces the frequency of intra-tumoral dendritic cells, which are crucial for T cell activation. The current study provides insights into the immunological events involved in the TBI/IL-2 combination in ACT.

## 1. Introduction

Adoptive T cell therapy (ACT) transfers tumor-specific T cells into cancer patients to augment the extent of cellular immune responses against the tumor. While it has shown promising results in some cancers, the therapeutic efficacy has been limited in most solid cancers [1,2,3,4]. To design effective strategies in ACT, critical immunological factors that affect the therapeutic outcome must be defined.

Lymphodepletion pre-conditioning is the most common regimen that is paralleled by T cell transfer to increase therapeutic efficacy. Pre-conditioning is known for inducing various features, such as the expansion of “space” for grafted T cells [5,6], depletion of cytokine sinks [7,8], and elimination of immune-suppressive cells [9]. Due to the diverse biological consequences caused by pre-conditioning, factors that drive pro- and anti-tumoral effects in ACT are difficult to identify. Studying pre-conditioning after “variable reduction,”, e.g., the use of a *Rag1* knock-out mouse strain, which is permanently lymphodepleted, may be an option to define factors that are not related to the effect of a large lymphoid space and endogenous T cells.

The administration of homeostatic cytokines, such as interleukin-2 (IL-2), is frequently used in ACT as a post-conditioning regimen [10]. It increases the persistence of transferred T cells by sustaining homeostatic signals during the therapy. Notably, interleukins often exhibit pleiotropy and can contribute to both pro- and anti-tumoral effects [11,12,13,14]. Therefore, the consequence of a certain post-conditioning may be varied depending on the immunological context that is induced by the pre-conditioning. To examine the feasibility of a combination of certain pre- and post-conditioning regimens in terms of synergy, a direct comparison of each regimen and combination should be conducted.

Here, we demonstrate the effect of total body irradiation (TBI) pre-conditioning and IL-2 treatment post-conditioning in ACT of melanoma. By using lymphodepleted *Rag1* knock-out mice, we focus on investigating the immunological changes induced by TBI. Additionally, the current study shows the consequences of the TBI/IL-2 combination regarding therapeutic outcomes and immunological profiles in lymphoid and tumor tissues. These findings will benefit endeavors to develop effective ACT strategies.

## 2. Materials and Methods

### 2.1. Mouse and Cell Line

*Rag1* knock-out (B6.129S7-*Rag1^tm1Mom^*/J) and Thy1.1 pmel-1 transgenic mice (B6.Cg-*Thy1^a^*/Cy Tg[TcraTcrb]8Rest/J; expressing a transgenic T cell receptor [TCR] specific for gp100_25–33_ and the congenic Thy1.1 antigen) were purchased from the Jackson Laboratory (JAX; Bar Harbor, ME, USA). All mice were maintained under specific-pathogen free conditions at the animal facility of the National Cancer Center in Korea. The B16-F10 melanoma cell line was purchased from ATCC (Manassas, VA, USA).

### 2.2. CD8^+^ T Cell Activation

To prepare melanoma-reactive T cells, CD8^+^ T cells were isolated from the lymph nodes and spleen of Thy1.1 pmel-1 mice using mouse CD8a Microbeads (Miltenyi Biotec, Inc., Auburn, CA, USA) according to the manufacturer’s instructions. The cells were stimulated with 5 μg/mL human gp100_25–33_ peptide (KVPRNQDWL; Peptron, Daejeon, Korea) in RPMI1640 media containing 10% FBS, antibiotics, and 5% of CD8-depleted splenocytes. Cells were used in the adoptive transfer experiment 2 days after the stimulation.

### 2.3. Adoptive Cell Transfer Model

On day 0, 2 × 10^5^ B16-F10 cells were injected subcutaneously into the back of *Rag1* knock-out mice. Three days later, mice were exposed to nonmyeloablative (4 Gy) TBI using an X-RAD 320 (Precision X-Ray, Inc., North Branford, CT, USA). On day 5, activated CD8^+^ T cells were intravenously injected into the mice via the lateral tail vein. Recombinant human IL-2 (10,000 IU; Novartis, Basel, Switzerland) was intraperitoneally administered daily for 3 days. Mice were routinely monitored for tumor growth and survival. Tumors were measured using calipers and the volume was calculated as: 1/2 × length × width × height. Mice were euthanized when the tumor volume reached 2500 mm^3^ or the animals displayed specific signs of illness (e.g., severe weight loss and tumor ulceration).

### 2.4. Flow Cytometry

On day 14, lymph node, spleen, and tumor tissues were collected from euthanized mice for the subsequent analyses. A single cell suspension of lymphoid tissues was prepared by gentle disruption of the inguinal tumor draining lymph nodes (TdLNs) and spleen, followed by filtration through a 40 μm nylon cell strainer (Falcon, NY, USA). Cells were treated with red blood cell lysis buffer (eBioscience, San Diego, CA, USA) before the analysis.

A tumor tissue-derived single cell suspension was prepared using a Tumor Dissociation Kit (Miltenyi Biotec, Inc., Auburn, CA, USA) according to the manufacturer’s instruction. Tumor tissues were cut into small pieces and transferred into the gentleMACS C tube containing the enzyme mix provided by the manufacturer. The tube was mounted on a gentleMACS dissociator (Miltenyi Biotec, Inc., Auburn, CA, USA) to dissociate the tumor tissue. The final single cell suspension was obtained by filtration through a 40 μm nylon cell strainer.

A single cell suspension was pre-incubated with the mouse Fc blocker for 5 min and further stained with fluorochrome-conjugated antibodies. Anti-MHC-Ⅱ:FITC, anti-CD8b:PE-Cy7, anti-CD206:BV421, anti-F4/80:BV510, anti-CD11c:BV655, anti-NK1.1:BV711 (Biolegend, San Diego, CA, USA), anti-PD-1:PE, anti-Ly6G:PE-CF594, anti-Ly6C:APC-Cy7, anti-CD80:BV786, anti-CD11b:BUV395, anti-Thy1.1:BUV737, and Fixable Viability Stain 700 (BD Biosciences, Franklin Lakes, NJ, USA) were used in the staining process according to the manufacturer’s instruction. In detail, the cells were washed twice with phosphate-buffered saline (PBS) and stained with Fixable Viability Stain 700 at 4 °C in the dark for 30 min. Afterward, they were washed once with staining buffer (PBS containing 2% FBS and 0.2% sodium azide) and stained with relevant fluorochrome-conjugated antibodies at 4 °C in the dark for 30 min. The stained cells were washed twice with staining buffer and fixed with PBS containing 1% paraformaldehyde overnight and subjected to flow cytometry analysis. Flow cytometry analysis was performed using LSRFortessa (BD Biosciences, Franklin Lakes, NJ, USA), and the data were analyzed using FlowJo (Tree Star, Inc., Ashland, OR, USA).

### 2.5. Statistical Analysis

All statistical data were analyzed in Prism v5.01 GraphPad (La Jolla, CA, USA). A two-tailed unpaired Student’s t-test was used in the comparison of cell counts and frequencies. The log-rank (Mantel-Cox) test determined the significance of the difference in survival rates. *p* values less than 0.05 were considered significant, which is designated with asterisks (* *p* < 0.05; ** *p* < 0.01; *** *p* < 0.001).

## 3. Results

### 3.1. TBI and IL-2 Treatment Independently Improves the Efficacy of Adoptive T Cell Therapy

**Figure 1:**
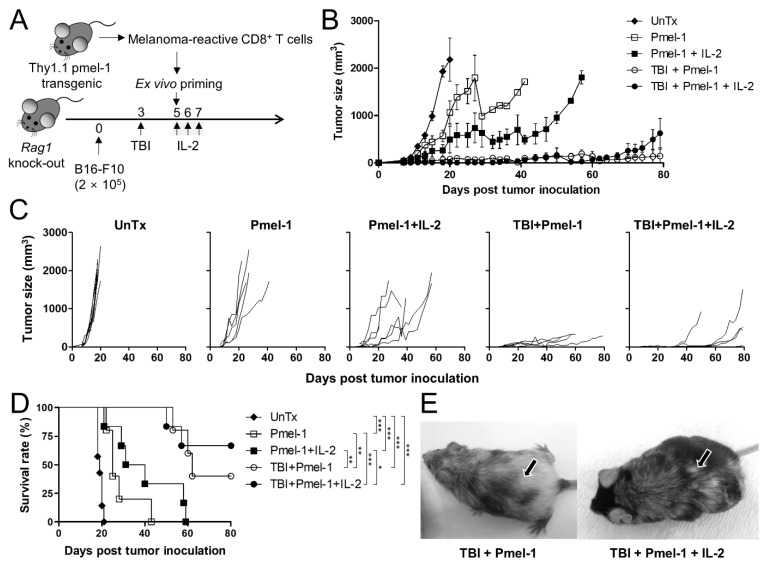
Anti-melanoma effect of total body irradiation and interleukin-2 treatment in adoptive T cell therapy. (**A**) Schematic drawing of the experiment. *Rag1* knock-out mice were subcutaneously inoculated with B16-F10 melanoma and treated with activated Pmel-1 as a form of adoptive T cell therapy. Pmel-1 stimulated for 2 days was administered into the mice on day 5. On day 3, some mice were exposed to 4 Gy total body irradiation (TBI). The interleukin-2 (IL-2) treatment group was injected daily (intraperitoneally) with 10,000 IU IL-2 on day 5 to day 7. (**B**) Tumor growth rate measured for 100 days. Each symbol and error bar indicate the mean and standard error of the mean (s.e.m.) of the tumor size in the same group. (**C**) Tumor growth rate of each mouse is indicated. (**D**) Kaplan–Meier curves showing the survival rate of the mice. (**E**) Representative images of the surviving mice in the TBI + Pmel-1 and TBI + Pmel-1 + IL-2 groups on day 80. Arrows indicate the tumor inoculation sites. UnTx (untreated) group, n = 7 mice; Pmel-1 and TBI + Pmel-1 groups, n = 5 mice per group; Pmel-1 + IL-2 and TBI + Pmel-1 + IL-2 groups, n = 6 mice per group. ns, not significant; * *p* < 0.05; ** *p* < 0.01; *** *p* < 0.001.

We investigated the therapeutic effect of TBI and/or IL-2 treatment in ACT of melanoma. The melanoma cell line B16-F10 was subcutaneously inoculated on the back of the *Rag1* knock-out mice, which are deficient in T and B cells. Pre-conditioning was conducted with 4 Gy TBI at 2 days before adoptive T cell transfer. Ex vivo primed Thy1.1 pmel-1 CD8^+^ T cells (Pmel-1) were infused on day 5, and some mice were treated daily with IL-2 for 3 days as a post-conditioning regimen (Figure 1A).

Mice treated with Pmel-1 alone were able to control tumor growth until 10 days but then rapidly lost the tumor-suppression effect (Figure 1B,C). More than half of the mice treated with both Pmel-1 and IL-2 displayed well-controlled tumor growth until 20 days. The TBI/Pmel-1 treatment further increased the efficacy, extending the period to more than 30 days. Intriguingly, the combined TBI, Pmel-1, and IL-2 treatment did not improve the therapeutic effect compared to the TBI/Pmel-1 treatment. Regardless of IL-2 treatment, tumors began to grow in all mice in the two groups 1 month after tumor inoculation. Survival of the mice was also improved following TBI treatment independently of IL-2 treatment (Figure 1D). Notably, the mice that survived more than 30 days presented with vitiligo as a symptom. One third of the Pmel-1/IL-2 treatment group and all mice in the TBI-treatment groups displayed vitiligo at 50 days post therapy (Figure 1E).

These results show that the transferred Pmel-1 increased the therapeutic efficacy particularly when it was accompanied by TBI. The effect was confirmed by suppressed tumor growth, increased survival rate, and the presentation of vitiligo, a positive prognostic factor [15,16]. Although the extent of efficacy was lower than with TBI, IL-2 treatment enhanced the anti-melanoma effect of Pmel-1. However, despite expecting the highest anti-tumor effect with the TBI/IL-2 combination, no additive effect was observed in the treated animals.

### 3.2. TBI/IL-2 Combination Increases the Proportion of Transferred T Cells in Lymphoid Tissues

Considering that the *Rag1* knock-out strain lacks endogenous T cells that drive strong adaptive immune responses, the key player of this model is likely to be the transferred Pmel-1. To elucidate the reason why the TBI/IL-2 combination had no additive effect, we investigated the Pmel-1 changes in these animals.

**Figure 2:**
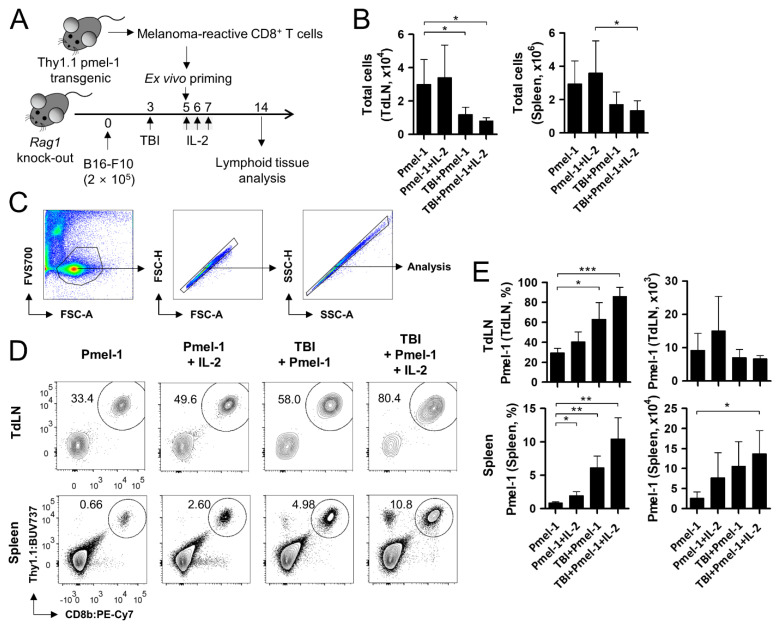
Analysis of adoptively transferred T cells in lymphoid tissues. (**A**) Schematic drawing of the experiment. Adoptive T cell transfer, total body irradiation (TBI), and interleukin-2 (IL-2) treatment were conducted as in Figure 1A. On day 14, the inguinal tumor-draining lymph nodes (TdLNs) and spleen were collected and analyzed. (**B**) Total count of the collected cells in the TdLNs and spleen. (**C**) Gating strategy of flow cytometry analysis. Among the acquired cell data, viable cells were gated in the FSC-A/FVS700 plot. Singlets were further gated in the FSC-A/FSC-H and SSC-A/SSC-H plots prior to the analyses of relevant markers. (**D**) Representative flow cytometry images showing Pmel-1 in the TdLNs and spleen. (**E**) Calculated frequency (left) and cell count (right) of Pmel-1 in lymphoid tissues. Pmel-1 and Pmel-1 + IL-2 groups, n = 3 mice per group; TBI + Pmel-1 and TBI + Pmel-1 + IL-2 groups, n = 5 mice per group. Each bar indicates the mean and standard deviation (s.d.) of each group. * *p* < 0.05; ** *p* < 0.01; *** *p* < 0.001.

On day 14, when the subjects displayed an active short-term immune response, the cell population in the TdLN and spleen was collected and analyzed (Figure 2A). First, we examined the magnitude of TBI-induced lymphodepletion and IL-2-induced lymphoproliferation. The total cell count of the TdLN significantly decreased when ACT was paralleled by TBI. Compared to the group with the sole Pmel-1 transfer, the cell count of the TBI/Pmel-1-treated group was reduced by about 50% in the TdLN and spleen (Figure 2B). The addition of IL-2 was insufficient to recover the TBI-induced cell destruction. Next, we checked the graft rate of Pmel-1 regarding the cell frequency modified by TBI and/or IL-2 treatment using flow cytometry (Figure 2C,D). TBI significantly increased the proportion of Pmel-1 in the TdLN and spleen (Figure 2D,E), indicating that the TBI-induced cellular space was refilled with the transferred Pmel-1. The increased proportion was mainly due to the depletion of other tissue-resident cells in response to TBI, since there was only minor increase in the total Pmel-1 count in these tissues (Figure 2E). IL-2 treatment also affected the proportional increase in Pmel-1 in the absence and presence of TBI.

Taken together, these data show that although TBI pre-conditioning and IL-2 treatment did not increase the genuine cell number of the transferred Pmel-1 in lymphoid tissues, the increased frequency implied an additive effect of the combination on the immunological profile of the subjects. At the same time, however, these results do not provide a clue to the absence of an additive effect between the TBI and IL-2 treatments.

### 3.3. TBI/IL-2 Combination Increases Tumor-Infiltrating Pmel-1 While Decreasing the Activation Intensity

**Figure 3:**
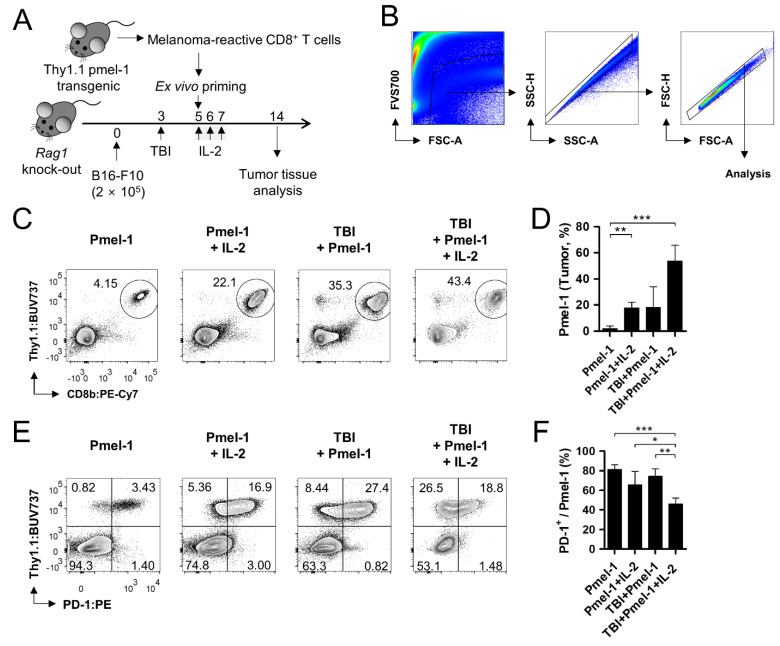
Analysis of adoptively transferred T cells in tumor tissues. (**A**) Schematic drawing of the experiment. Adoptive T cell transfer, total body irradiation (TBI), and interleukin-2 (IL-2) treatment were conducted as in Figure 1A. On day 14, the inoculated tumor tissue was dissociated into a single cell suspension and analyzed using flow cytometry. (**B**) Gating strategy of flow cytometry analysis. (**C**) Representative flow cytometry images showing tumor-infiltrating Pmel-1. Among the acquired cell data, viable cells were gated in the FSC-A/FVS700 plot. Singlets were further gated in the SSC-A/SSC-H and FSC-A/FSC-H plots prior to the analyses of relevant markers. (**D**) Determined frequency of Pmel-1 in tumor tissue. (**E**) Representative flow cytometry images showing PD-1 expression on Pmel-1. (**F**) Determined frequency of PD-1^+^ cells within the Pmel-1 population. Pmel-1 and Pmel-1 + IL-2 groups, n = 3 mice per group; TBI + Pmel-1 and TBI + Pmel-1 + IL-2 groups, n = 4 mice per group. Each bar indicates the mean and standard deviation (s.d.) of each group. * *p* < 0.05; ** *p* < 0.01; *** *p* < 0.001.

The quantitative feature of tumor-infiltrating T cells accounts for the magnitude of anti-tumor responses. Therefore, we focused on the tumor-infiltrating Pmel-1 to identify the factor associated with the reduced additive effect in the TBI/IL-2 combination. Using the same condition as was used for the lymphoid tissue analysis, the tumor tissues on day 14 were dissociated into single cells and analyzed using flow cytometry (Figure 3A,B).

The TBI increased the frequency of the tumor-infiltrating Pmel-1 as was similarly observed in the lymphoid tissues (Figure 3C,D). Notably, IL-2 and TBI contributed to the proportional change, respectively, and the additive effect between the treatments was clearly observed in the result. We found that nearly half of the tumor-infiltrating live cells were Pmel-1 in the TBI/IL-2 combination group. Considering the differences in the tumor-reactive Pmel-1 frequency in the lymphoid and tumor tissues, there being no difference in the therapeutic effect between the TBI and TBI/IL-2 groups was contradictory (Figure 1B,C). Intriguingly, the following qualitative analysis of the tumor-infiltrating Pmel-1 resulted in an unexpected finding. We checked PD-1 expression level on Pmel-1 among the groups and found that TBI group had higher proportion of PD-1^+^ T cells compared to the TBI/IL-2 combination (Figure 3E,F). PD-1 is one of the representative inhibitory receptors in T cells, and its expression at early timepoints (hours to days after antigen encounter) reflects appropriate activation [17]. Given that the tumor-infiltrating Pmel-1 was analyzed on day 14, which was 9 days after adoptive transfer, the significant reduction in the PD-1^+^ proportion by the TBI/IL-2 combination implied insufficient activation. To elucidate the reason for this consequence, we investigated the detailed immunological features in the following experiments.

### 3.4. TBI and IL-2 Combination Alters the Immune Cell Landscape in Melanoma-Bearing Rag1 Knock-Out Mice

The purpose of pre-conditioning is to remove immune-suppressive cells before the adoptive transfer of tumor-reactive T cells. As all pre-conditioning regimens currently in use are not target-specific, diverse immune cell subsets that are not immune-suppressive are also likely to be affected by the regimens. Therefore, we sought to identify significant changes in the subsets that may have roles in anti- and pro-tumoral effects, by which the absence of the TBI/IL-2 combination additive effect can be explained.

**Figure 4:**
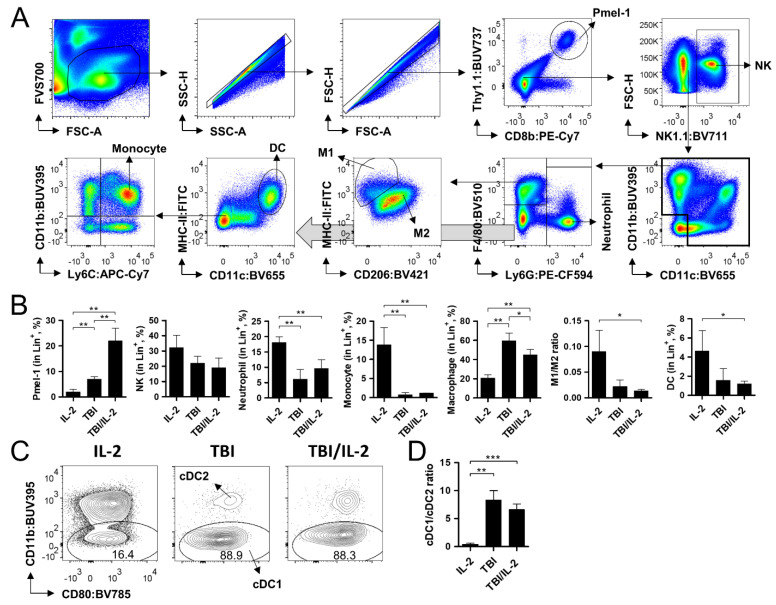
Analysis of the immune cell population in the spleen after adoptive T cell therapy. Adoptive T cell transfer, total body irradiation (TBI), and interleukin-2 (IL-2) treatment were conducted as in Figure 1A. On day 14, the spleen was collected and subjected to multi-parameter flow cytometry analysis. (**A**) Gating strategy of the analysis. Among the acquired cell data, viable cells were gated in the FSC-A/FVS700 plot. Singlets were further gated in the SSC-A/SSC-H and FSC-A/FSC-H plots prior to the analyses of relevant markers. First, Pmel-1 (CD8b^+^/Thy1.1^+^) and natural killer cells (NK; NK1.1^+^) were defined among the singlets. Myeloid cells (CD11c^+^ and/or CD11b^+^) were divided into macrophages (F4/80^+^), neutrophils (Ly6G^+^), and other cells (double negative) in the Ly6G/F4/80 plot. After dendritic cells (DCs; CD11c^+^/MHC-II^+^) were excluded from the double negative subset, monocytes (Ly6C^+^/CD11b^+^) were defined. Types 1 and 2 macrophages (M1 and M2) were gated in the CD206/MHC-II plot. (**B**) Frequency of diverse immune cell subsets in the spleen. The frequency of Pmel-1, NKs, neutrophils, macrophages, DCs, and monocytes among lineage marker-positive cells (Lin^+^; positive for Thy1.1, NK1.1, CD11b, and/or CD11c) was calculated. For M1 and M2, the ratio is indicated. (**C**) Representative flow cytometry images showing conventional types 1 and 2 DCs (cDC1 and cDC2) within the DC population. (**D**) Calculated ratio between cDC1 and cDC2. Pmel-1 + IL-2 and TBI + Pmel-1 groups, n = 3 mice per group; TBI + Pmel-1 + IL-2 group, n = 4 mice per group. Each bar indicates the mean and standard deviation (s.d.) of each group. * *p* < 0.05; ** *p* < 0.01; *** *p* < 0.001.

**Figure 5:**
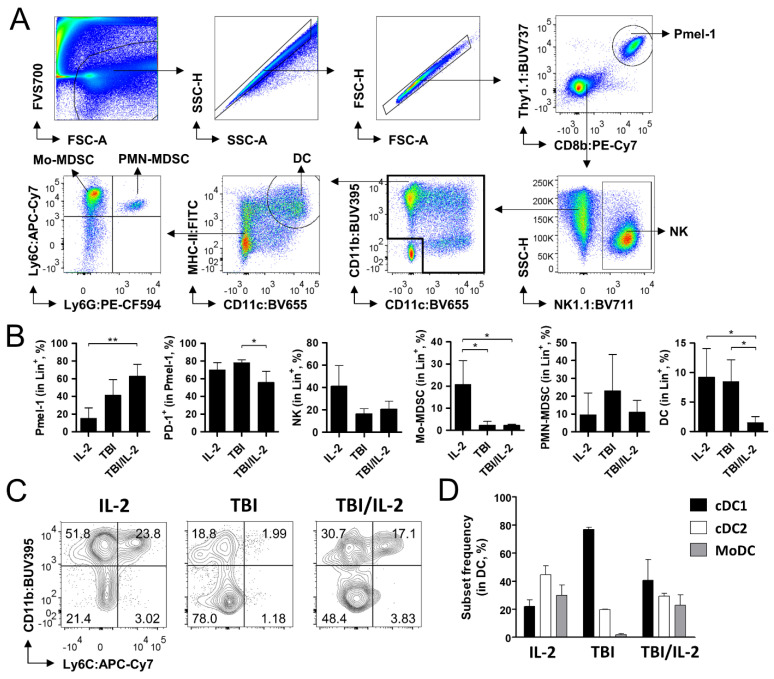
Analysis of the immune cell population in the tumor after adoptive T cell therapy. Adoptive T cell transfer, total body irradiation (TBI), and interleukin-2 (IL-2) treatment were conducted as in Figure 1A. On day 14, tumor tissue was collected and subjected to multi-parameter flow cytometry analysis. (**A**) Gating strategy of the analysis. Among the acquired cell data, viable cells were gated in the FSC-A/FVS700 plot. Singlets were further gated in the SSC-A/SSC-H and FSC-A/FSC-H plots prior to the analyses of relevant markers. First, Pmel-1 (CD8b^+^/Thy1.1^+^) and natural killer cells (NK; NK1.1^+^) were defined among the singlets. Dendritic cells (DCs; CD11c+/MHC-II+) were defined among CD11c- and/or CD11b-expressing myeloid cells. Monocytic myeloid-derived suppressor cells (Mo-MDSCs) and polymorphonuclear MDSC (PMN-MDSC) were gated in the Ly6G/Ly6C plot. (**B**) Frequency of diverse immune cell subsets in the tumor. The frequency of Pmel-1, NKs, DCs, monocytic myeloid-derived suppressor cells (Mo-MDSCs), and polymorphonuclear MDSC (PMN-MDSC) among lineage marker-positive cells (Lin^+^; positive for Thy1.1, NK1.1, CD11b, and/or CD11c) was calculated. For Pmel-1, the PD-1-positive proportion is additionally indicated. (**C)** Representative flow cytometry images showing monocyte-derived DCs (MoDCs) and conventional types 1 and 2 DCs (cDC1 and cDC2) within the DC population. (**D**) The frequency of cDC1, cDC2, and MoDC is indicated. Pmel-1 + IL-2 and TBI + Pmel-1 groups, n = 3 mice per group; TBI + Pmel-1 + IL-2 group, n = 4 mice per group. Each bar indicates the mean and standard deviation (s.d.) of each group. * *p* < 0.05; ** *p* < 0.01.

The *Rag1* knock-out strain lacks B and T cells, and its immune cell population mainly consists of natural killer (NK) cells and other myeloid lineage cells. We investigated TBI/IL-2-induced changes in these subsets by conducting multiparametric flow cytometry analysis of the cells isolated from spleen and tumor tissue. Tumor tissues were analyzed on day 14 when the size was relatively small (<100 mm^3^) and ideal for investigating immune cell reconstitution. We defined several immune subsets from the spleen and tumor by using CD8b, Thy1.1, PD-1, NK1.1, CD11b, CD11c, MHC-II, CD80, Ly6G, Ly6C, F4/80, and CD206 antibodies (Figure 4A and Figure 5A). The frequency of each subset within the lymphoid/myeloid cells (Lin^+^; positive for Thy1.1, NK1.1, CD11b, and/or CD11c) was compared among the groups. As observed in the previous results (Figure 2E and Figure 3D), the ratio of Pmel-1 increased in the lymphoid and tumor tissues when the animals were treated with the TBI/IL-2 combination (Figure 4B and Figure 5B). The decreased proportion of PD-1^+^ Pmel-1 in the TBI/IL-2-treated tumor was also consistent with the former data (Figure 3F and Figure 5B), indicating that these groups have an immunological status equivalent to that of the experimental setting shown in Figure 2 and Figure 3. In the spleen, the presence of TBI significantly decreased the frequency of neutrophils and monocytes regardless of additional IL-2 treatments (Figure 4B). In contrast to these subsets, TBI increased the ratio of splenic macrophages particularly enriching the CD206^+^ M2 subtype as observed in a previous report [18]. Notably, dendritic cells (DCs), a crucial component in T cell activation, showed a predominant alteration in the ratio of conventional DC types 1 and 2 (CD11b^−^ cDC1 and CD11b^+^ cDC2) sub-populations. Compared to the IL-2 treatment group, the TBI and TBI/IL-2 treatment groups increased the cDC1 to cDC2 ratio from 0.39 to 8.29 and 6.61 (Figure 4C,D).

In the tumor tissue, we observed substantial changes in the frequency of DCs among the tumor-infiltrating immune cells and alteration in the subset constitution, which consisted of monocyte-derived DCs (Ly6C^+^ MoDC), cDC1, and cDC2 (Figure 5B–D). Myeloid-derived suppressor cells (MDSCs), which are well-known drivers in immune-suppression [19,20,21,22], showed a decrease in monocytic MDSCs (Ly6Chi Ly6G^−^ Mo-MDSCs) after TBI treatment, while no significant change was shown in the polymorphonuclear MDSCs (Ly6C^+^ Ly6G^+^ PMN-MDSCs) among the groups.

Taken together, we observed the effect of TBI and IL-2 treatments on immune cell populations in lymphoid and tumor tissues. TBI not only altered the frequency of DCs but also reshaped the subset distribution in spleen and tumor tissue. Notably, compared to the sole TBI treatment, the TBI/IL-2 combination significantly decreased tumor-infiltrating DCs, while particularly reducing the population size of the cDC1 subset. Given that cDC1 plays an important role in tumor-specific T cell activation [23,24], these features may affect Pmel-1 activity in TBI/IL-2-treated mice. The decrease in neutrophils and monocytes in the spleen and Mo-MDSCs in the tumor after TBI treatment are other factors that can contribute to the anti-tumor efficacy in these groups, though no significant difference was observed between the TBI and TBI/IL-2 treatment groups.

## 4. Discussion

In the current study, we investigated the combination of TBI pre-conditioning and IL-2 post-conditioning regarding the therapeutic effect and immunological profile in ACT of murine melanoma. As expected from the previous studies that described the anti-tumor effect of these conditioning regimens [10,25], both TBI and IL-2 treatments improved the anti-tumor activity and survival rate of Pmel-1-infused mice. Intriguingly, the combination of regimens failed to improve the therapeutic outcome in contrast to the expected synergy between TBI and IL-2 conditioning. We found that while the frequency of Pmel-1 increased in the lymphoid and tumor tissues, the activation intensity of tumor-infiltrating Pmel-1 was reduced after the combination. Multiparametric flow cytometry analysis revealed alterations in the immune cell populations by the TBI/IL-2 combination. Modification of the frequencies of spleen-resident neutrophils, monocytes, macrophages, and tumor-infiltrating Mo-MDSCs were associated with TBI treatment regardless of IL-2 treatment. Notably, by comparing the TBI and TBI/IL-2 treatment groups, we identified the quantity and subset distribution of DCs as potential factors that affect Pmel-1 activation. A decrease in the frequencies of whole DCs and the cDC1 subset in tumor tissue suggests an explanatory mechanism of how the TBI/IL-2 combination reduces the additive effect in ACT of murine melanoma.

DCs play a key role in the ‘Cancer–Immunity Cycle’ by activating T cells with tumor-specific TCRs [26]. Notably, the effect is context-dependent as the sub-populations consist of cDC1, cDC2, MoDC, and plasmacytoid DC (pDC), which are involved in various pathways of immune activation and suppression [27,28]. cDC1 is crucial in activating tumor-specific CD8^+^ T cells [23,24], whereas cDC2 promotes the activation of CD4^+^ T cells and has been known for driving anti-tumoral activities [29,30]. MoDC, which is frequently observed in tumors, is implicated in immunosuppression [31,32]. Since the current study setting lacks tumor-specific CD4^+^ T cells, an increased cDC1 to cDC2 ratio induced by TBI in the spleen was a better prognostic factor. Most importantly, the altered DC subset distribution in the tumor is likely to account for reduced Pmel-1 activation, since the mice that underwent TBI without IL-2 treatment showed the highest frequency of cDC1 and the lowest frequency of MoDC. Nevertheless, further study must be conducted to corroborate the hypothesis considering the controversies in MoDC function [33]. Additionally, considering the inhibitory role of PD-1 even in the early phase of T cell activation [17], an “insufficiently” activated Pmel-1 by the TBI/IL-2 combination could likely function better than a sufficiently activated Pmel-1 in the TBI group. A detailed longitudinal study may help explain the genuine consequences of the enhanced activation by TBI.

In addition to DCs, we found that TBI changed the landscape of diverse immune cells that drive pro- and/or anti-tumoral functions in lymphoid and tumor tissues. Neutrophils were a subset significantly affected by TBI in the spleen. A recent study by Veglia et al. showed that spleen-resident neutrophils developed PMN-MDSC-like characteristics after tumor inoculation in a mouse model [34]. Despite no significant difference in tumor-infiltrating PMN-MDSCs among the groups, we observed a 2- to 3-fold reduction in splenic neutrophils after TBI. Considering the immune-suppressive activity of PMN-MDSCs in cancer [20,21], this may account for the enhanced therapeutic efficacy by TBI. A reduction in the frequency of monocytes paralleled by the increase in splenic macrophages was another feature of TBI-treated mice. Given that the cells mainly consisted of M2 macrophages, which were associated with immune suppression [35], TBI-induced sterile inflammation and the subsequent enrichment of the M2 subtype could be a factor that drives the pro-tumoral effect in TBI treatment. Mo-MDSCs, a strong mediator of immune suppression [19,22], indicated a ~10-fold reduction in the frequency following TBI conditioning. Considering that ~20% of lymphoid/myeloid cells in the tumor were Mo-MDSCs in the group without TBI treatment, this alteration was likely to increase the anti-tumor effect in the current study.

*Rag1* knock-out mice, the only strain used throughout this study, lack main components in the lymphoid lineage, such as T and B cells. Therefore, we could investigate the changes in various immune-related components apart from the dominant effect of the lymphodepleted space and endogenous T cells. Concomitantly, however, this means that the results found in the current study are not directly translated into immune-competent settings of other mouse models and human cancers. The key factor in this issue is the absence of T cells, which play essential roles in pro- and anti-tumoral activities. Endogenous CD8^+^ T cells that have polyclonal TCRs are a component that contributes to anti-tumor activity through the ‘Cancer–Immunity Cycle’ [26]. In the cycle, the killing of cancer cells by Pmel-1 results in the release of various tumor antigens and subsequently primes other endogenous T cells with a diverse TCR repertoire in lymphoid tissues. The use of the *Rag1* knock-out strain and Pmel-1 in this study removed the benefit of a diverse arsenal of endogenous T cells, thus promoting cancer immune escape from single epitope-specific Pmel-1. Additionally, the lack of lymphoid cells likely widened the disparity in the tissue-intrinsic profile between the LN and spleen. For instance, the current setting presented that the proportion of non-Pmel-1 was higher in the spleen than in the lymph node (Figure 2E; 20–70% in the TdLN and 90–99% in the spleen). This feature likely resulted in the increased Pmel-1 count in the TBI-treated spleen, which was in contrast with TdLN, by providing additional supplementary signals from myeloid subsets. Regulatory T cells (Tregs), a subset of CD4^+^ T cells that maintain immune homeostasis and self-tolerance, are a strong suppressor of tumor-specific T cell responses and are thus an important pro-tumoral component in the immune system [36,37]. Since Tregs are absent in the current study, the effect of TBI and IL-2 on Pmel-1 may be different in the immune competent condition. These limitations should be carefully considered before interpreting the results.

TBI intensity determines the extent of myeloablation that the subject undergoes during ACT. Previous reports showed that the intensity of lymphodepletion correlated with the efficacy of ACT, since high intensity lymphodepletion removed endogenous cells with potential inhibitory activities [38]. However, high dose radiation around 10 Gy induces myeloablation, thus requiring CD34^+^ hematopoietic stem cell (HSC) transplantation [39]. Therefore, we used 4 Gy TBI, which causes nonmyeloablative lymphodepletion and dispenses with HSC transplantation. Although we observed various changes in the immune profiles after low dose TBI in this study, high dose TBI and an HSC graft may lead to different consequence regarding synergy with IL-2 because of the mechanistic disparity [39]. Other modifications such as local irradiation can also result in a better outcome as it targets tumor-infiltrating immune cells, which mostly consist of immune-suppressive populations. For instance, local tumor irradiation can spare splenic M1 macrophages with anti-tumor activity [40,41], which was depleted by TBI in the current study.

## 5. Conclusions

We showed that non-myeloablative TBI and IL-2 treatment independently contributed to ACT efficacy, whereas the combination failed to induce an additive effect between the regimens. The underlying factors related to this outcome included alterations in the DC sub-populations and insufficient activation of transferred Pmel-1. This highlights the importance of sufficient antigen presentation even if the tumor-specific T cells are abundant in the recipient. Lastly, other immunological changes induced by TBI provides insights into the development of an effective ACT strategy in future studies.

## Data Availability

All data supporting the findings of this study are available within the article.
